# Transcriptome profiling during mangrove viviparity in response to abscisic acid

**DOI:** 10.1038/s41598-018-19236-x

**Published:** 2018-01-15

**Authors:** Liwei Hong, Wenyue Su, Yuanye Zhang, Congting Ye, Yingjia Shen, Qingshun Q. Li

**Affiliations:** 1 Key Laboratory of the Ministry of Education for Coastal and Wetland Ecosystem, College of the Environment and Ecology, Xiamen University, Xiamen Fujian, 361102 China; 2 Graduate College of Biomedical Sciences, Western University of Health Sciences, Pomona, CA 91766 USA

## Abstract

Mangrove plants adapt to coastal tidal mudflats with specially evolved viviparity seed development. However, very little is known about the genetic and molecular mechanisms of mangrove viviparity. Here, we tested a hypothesis that plant hormone abscisic acid (ABA) plays a significant role in precocious germination of viviparous *Kandelia obovata* seeds by exogenous applications. Through transcriptome analysis of ABA treated seeds, it was found that ABA repressed mangrove fruit growth and development, and there were thousands of genes differentially expressed. As a result, dynamics of the pathways were dramatically altered. In particular, “Plant hormone signal transduction” and “MAPK signaling pathway” were represented significantly. Among differentially expressed genes, some key genes of ABA signal transduction were induced, while ABA biosynthesis genes were repressed. Take *ABI1* and *ABI2*, key negative regulators in ABA signal pathway, as examples, homologous alignment and a phylogenetic tree in various species showed that *ABI1* and *ABI2* are highly conserved among various species. The functional similarity of these genes was confirmed by transgenic work in *Arabidopsis*. Taken together, ABA inhibited mangrove viviparity, but mangroves developed a mechanism to prevent accidently increase of ABA in the harsh environment for maintaining viviparous reproductive strategy.

## Introduction

Mangroves are a group of trees and shrubs that grow in the intertidal zones of tropical and subtropical coastlines around the world. There are approximately 80 species of mangroves with genetic diversity derived from 20 plant families, and they have developed different morphological and physiological solutions to adapt harsh coastal conditions in a convergent and shared evolution^1,2^. Mangrove forest ecosystem plays an important role in estuaries and marine shorelines. For examples, mangrove forests stabilize the coastline, reducing erosion from storm surges, currents, waves, and tides, and the intricate root system of mangrove trees also makes these forests attractive for aquatic life and other organisms to seek food and shelter from predators. People benefit from mangrove ecosystems for foods and forest products. Despite their ecological and economic importance, mangrove forests are being destroyed at an alarming rate. It appears that 35% of the mangroves, of those areas for which data has been collected, have been destroyed^3^. The United Nations Environment Program and Hamilton estimate that shrimp farming causes about 25% destruction of mangrove forests^4,5^. Fortunately, many countries have made rules for biodiversity conservation^6^ in an attempt to reverse such a trend. Scientific understanding on how mangrove plants develop special features like viviparity to survive/adapt to their habitat could be helpful to guide the conservation planning and management of mangrove forest ecosystem resources.

Viviparity in flowering plants is defined as the continuous growth of the offspring whilst attached to the maternal plant without apparent dormant period^7,8^. Dormancy, which established during late embryo maturation in higher plants, ensures that seeds could overcome unfavorable conditions between drop-off from the mother plant and germination^9,10^. The transition of the seeds from dormancy to germination is a key physiological process during plant life-cycle. In contrast to orthodox seeds, which experience dormancy, drying and resting process^10,11^, many mangrove plant species have evolved a special mechanism to help their offspring survive in the harsh environment of tropical and subtropical estuaries and marine shorelines, such as high salinity, high temperature, high humidity, water logging, hypoxia and tidal waves. Seeds of some mangroves use vivipary mechanism thus do not enter dormancy nor desiccation, which is an adaptive feature to withstand the hostile environment and expand their populations at coastal tropical habitats.

In fact, there are three kinds of reproductive strategies in mangrove plants: vivipary (e.g. *Kandelia obovata* and *Rhizophora apiculata*)^12,13^, cryptovivipary (e.g. *Avicennia marina*)^14^ and non-vivipary (e.g. *Sonneratia alba*)^15^. Several studies have been performed to reveal morphological, biochemical, genetic and molecular causes of these phenomena^16–19^. Phytohormones, especially abscisic acid (ABA) and gibberellic acid (GA) play important roles in regulating the viviparous process^18,20^. It was reported that dynamic change of salt ions^21,22^ and water content^23^ during embryo and propagule development helps for adaption to its habitat. However, the molecular understanding of viviparous mechanisms in mangroves is relatively scarce. What is known is that vivipary in mangroves is a genetically programed process and it happens regardless the acute environmental conditions. This is in stark contrast to other viviparous germination of Arabidopsis, maize and rice where precocious germinations largely depend on the environmental conditions (high humidity and/or high temperature) or genetic mutations^24–26^.

ABA is one of the most important hormones in plants, which was known for its role in plant development and responses to environmental stresses^27,28^. Studies also show that ABA is required to establish seed dormancy in Arabidopsis and other seed plants^9,29,30^. ABA-deficient and ABA-insensitive mutants show partial loss of dormancy, and often lead to viviparous germination^25,31^. Application of an inhibitor of ABA synthesis, fluridone, at early stages of embryo maturation induces precocious germination^32,33^, while desiccation during mid-to-late embryogenesis is correlated with increased ABA concentration^34^. Exogenous application of ABA delays germination or even kills the seeds, and ABA hypersensitivity is associated with increased dormancy^30^. ABA levels are consistently lower during embryo development of viviparous mangrove than that of its non-viviparous sister species^18,35^. ABA accumulation in plants can also be induced by environmental stresses, such as salt, reactive oxygen species and high temperature^36–39^.

So far, more than 100 genes have been identified to be involved in ABA signaling^40,41^, including ABA-insensitive loci *ABI1*, *ABI2*^42^ as well as ABA-deficient loci *ABA1* and *ABA2* in Arabidopsis^43,44^. Among them, *ABI1* and *ABI2* encode a group of type 2 C protein phosphatases (PP2Cs) localized both in the cytoplasm and nucleus^45^, and act in a negative feedback regulatory loop of the ABA signal transduction pathway^46,47^. The gain-of-function mutants of *ABI1* and *ABI2* are ABA-insensitive and show reduced seed dormancy^42,48^, while disrupted expression of *ABI1* and *ABI2* results in increased sensitivity to ABA in post-germination growth assays^49^. *ABA1* (encodes a zeaxanthin epoxidase)^43^ and *ABA2* (encodes a cytosolic short-chain dehydrogenase/reductase)^44^ are two key enzymes playing important roles in ABA biosynthesis, mutants of which resulting in ABA deficiency.

The function of ABA in regulating fruit development and ripening^50–52^, as well as the finding that levels during embryo development of viviparous and non-viviparous mangrove plants are different^18,35^, suggest that ABA plays a role in viviparous embryo development of mangrove. However, there lacks of direct proof of this conclusion, and the internal molecular mechanism remains to be explored. In this study, we investigate the inhibition effects of exogenous ABA to the fruit development and transcriptional response of a true viviparous mangrove plant *K*. *obovata*. Our data show that ABA synthesis was suppressed under ABA treatment, but ABA signal transduction pathway was induced. In particular, key regulators of this pathway, *ABI1* and *ABI2*, two highly conserved genes in mangroves, were induced by exogenous ABA. We also demonstrated that, they have similar functions in transgenic tests in Arabidopsis. These results indicate that ABA inhibits viviparity in *K*. *obovata*, and there exists a negative regulatory loop for keeping viviparous reproductive strategy in mangroves.

## Results

### Responses of *K*. *obovata* fruit to exogenous ABA

It has been shown that ABA is required to establish seed dormancy and suppress seed germination in Arabidopsis and other seed plants^9,29,30^. ABA also plays a role in regulating fruit development and ripening^50–52^. In addition, ABA is kept in a lower level during embryo development of viviparous mangrove than that of its non-viviparous sister species^18,35^. All these findings suggest that ABA participates in embryo development of mangrove viviparous process.

**Figure 1:**
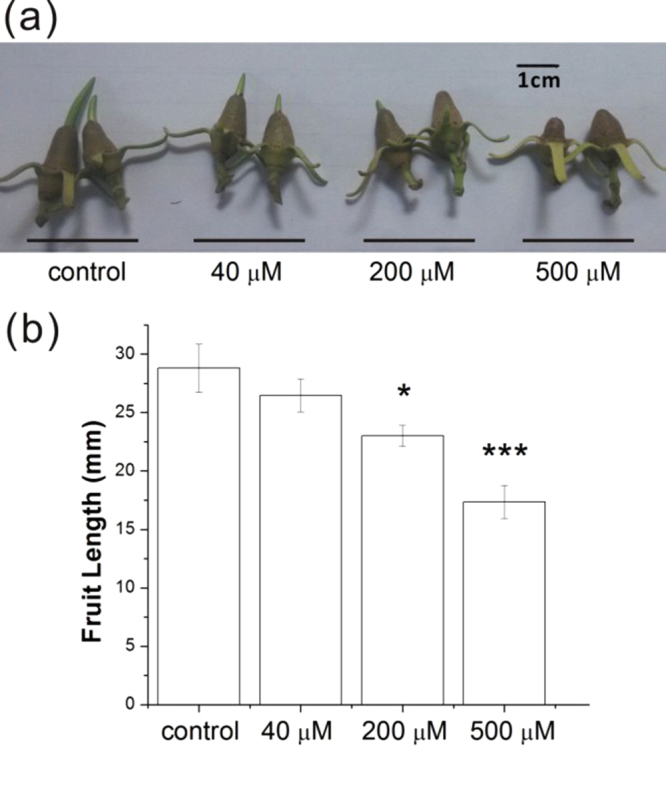
Effects of exogenous ABA on *K*. *obovata* fruit growth. (**a**) Fruit of *K*. *obovata* with or without exogenous ABA treatment. (**b**) Fruit length in (**a**). Mean ± SD values were determined from three replicates (n > 40). Asterisks represents statistical significance (Student’s t-test, * and ***P < 0.05 and 0.001, respectively).

*K*. *obovata* is a species of mangrove plant in the *Rhizophoraceae* family, mainly found in Southern China^12^. In order to understand the role of ABA in the mangrove viviparous process, three concentrations of 40, 200 and 500 μM ABA were used to treat the early stage fruits of *K*. *obovata*. The results showed that, compared to the Tween-20-treated group (control) and low concentration ABA-treated group (40 μM ABA), 200 and 500 μM ABA strongly repress fruit development and growth (Fig. 1a). The inhibition effect on fruit elongation enhanced with the increase of ABA concentration (Fig. 1b). The surface of fruit under 500 μM ABA treated was irregular, and some even malformed, indicating that ABA worked as a negative regulator in mangrove vivipary. However, ABA application at this concentration could not stop mangrove vivipary entirely.

### Global transcriptome analysis upon ABA treatment

To conduct transcriptome analysis of the influence of ABA on mangrove vivipary, we collected the embryo samples with or without 500 μM ABA treatments for deep sequencing by RNA-seq, which exhibited the most considerable distinctions. cDNA libraries constructed from the total RNA of embryos were subjected to deep sequencing performed on Illumina Hiseq 2500 platform.

**Figure 2:**
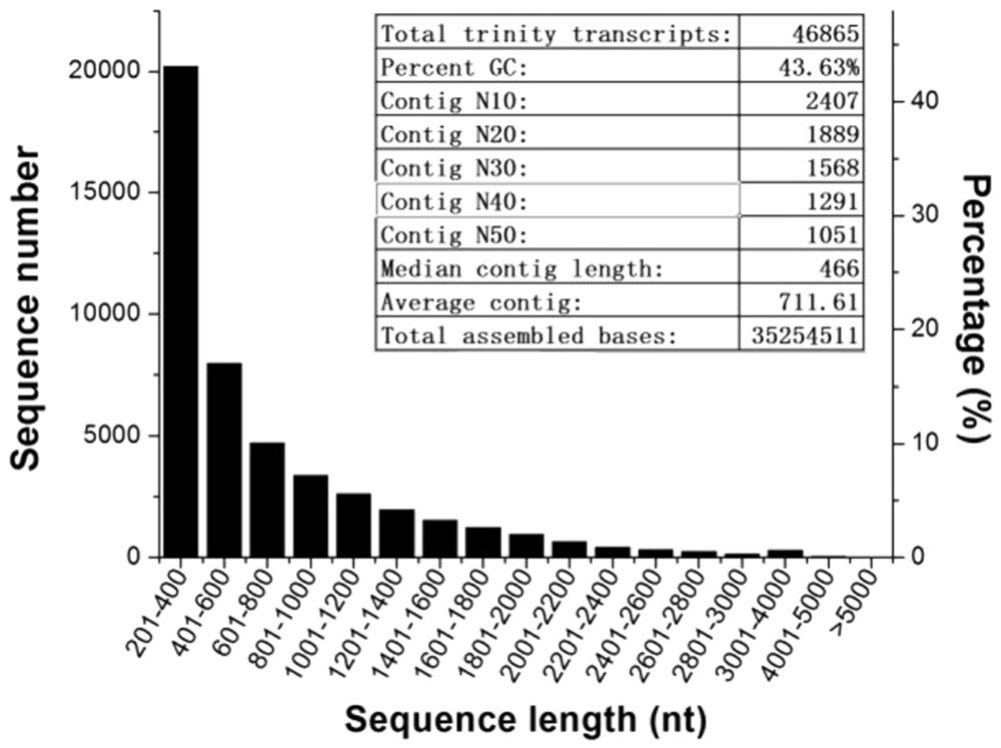
Distribution of transcripts length and contigs in the global transcriptome analysis of *K*. *obovata* propagules treated with and without 500 μM ABA.

The high-throughput sequencing generated 227,147,050 reads, a total raw dataset of 60.14 Gb. After trimming low quality sequences, sequencing adapters and ambiguous nucleotides, clean data with a total of 192,488,542 reads were obtained, and the read density was sufficient for the subsequent quantitative analysis of gene expression (Table S2). These high quality clean data were assembled into 46,865 transcripts, with an N50 length of 1,051 bp and an average size of 711 bp, as shown in Fig. 2. Assembled transcripts ranged from 201 to 8,420 bp. Transcripts of length 201–400 bp accounted for 43.12% of the total, those 401–1,000 bp for 34.32%, those 1,001–2,000 bp for 17.84%, and those >2,000 bp for 4.72% (Fig. 2).

### GO and KEGG analysis of all detected transcripts

**Figure 3:**
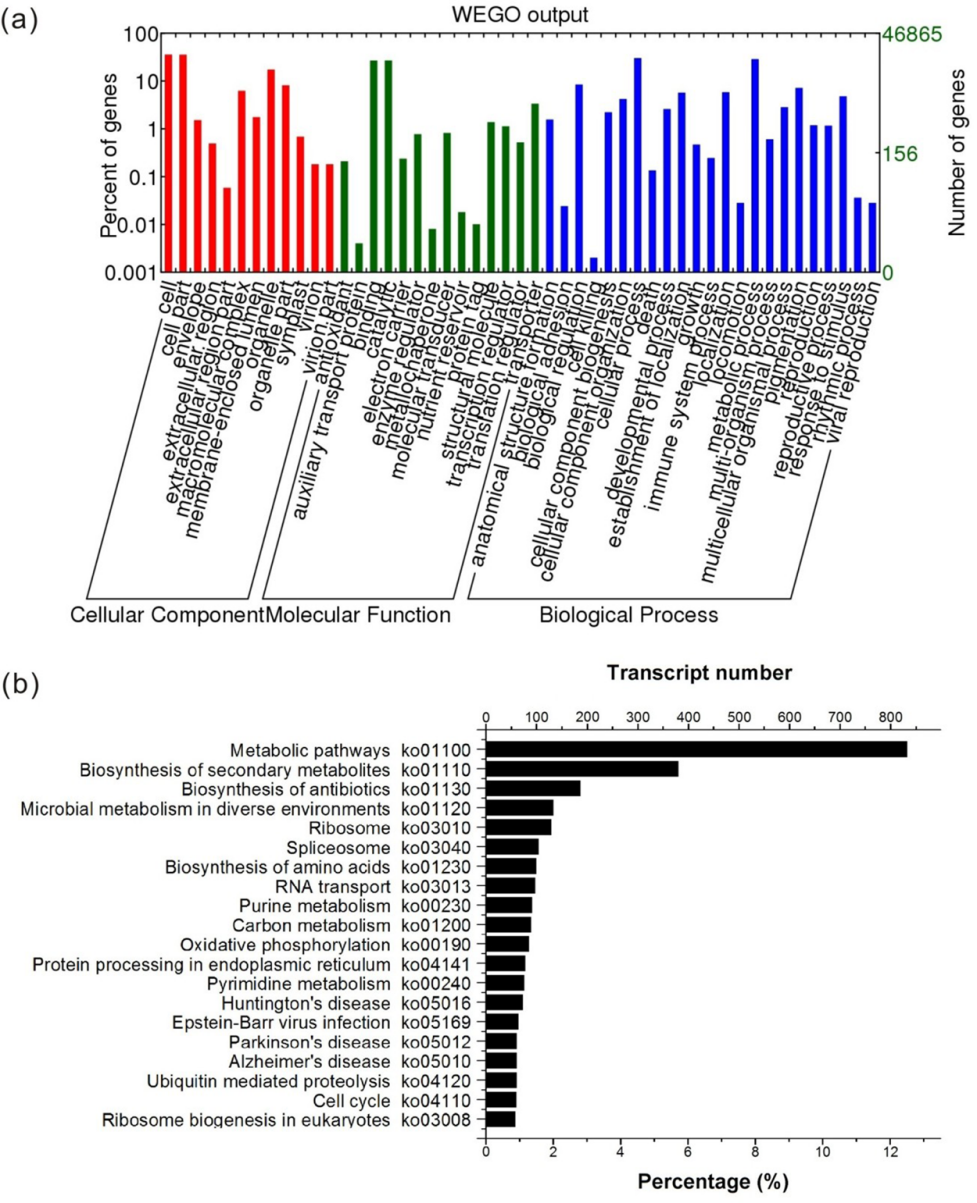
Gene Ontology and Kyoto Encyclopedia of Genes and Genomes enrichment of *K*. *obovata* transcriptome assembled by all samples. (**a**) GO enrichment of the *K*. *obovata* transcriptome. (**b**) KEGG analysis of the *K*. *obovata* transcriptome (top 20).

To functionally classify the transcriptome, all identified genes of *K*. *obovata* were classified into different functional categories using Blast2GO^53^, including biological processes, molecular functions, and cellular components. Out of all 46,865 genes of the assembled transcriptome, 26,849 transcripts could be successfully aligned to GO terms and classified into 49 functional groups, including 23 groups in biological process, 12 in cellular component, and 14 in molecular function (Fig. 3a). In the category of cellular component, the three main groups were “cell” (GO: 0005623, 17694 transcripts) and “organelle” (GO: 0043226, 8583 transcripts). For molecular functions, a large number of genes were involved in the “binding” (GO: 0005488, 13298 transcripts) and “catalytic activity” categories (GO: 0003824, 13352 transcripts). Within the class of biological process, “biological regulation” (GO: 0065007, 4186 transcripts) and “metabolic process” (GO: 0008152, 14239 transcripts) were predominant (Fig. 3a).

The total expressed genes were subjected to KEGG pathway enrichment analysis based on sequence similarity, and 6,664 transcripts were assigned to 365 KEGG pathways. The major pathways represented in the transcriptome of *K*. *obovata* embryos were “metabolic pathways” (ko01100, 832 transcripts), “biosynthesis of secondary metabolites” (ko01110, 380 transcripts), followed by “biosynthesis of antibiotics” (ko01130, 186 transcripts), “microbial metabolism in diverse environments” (ko01120, 133), “ribosome” (ko03010, 129), and “spliceosome” (ko03040, 104) (Fig. 3b).

### Differential gene expression before and after ABA treatment

**Figure 4:**
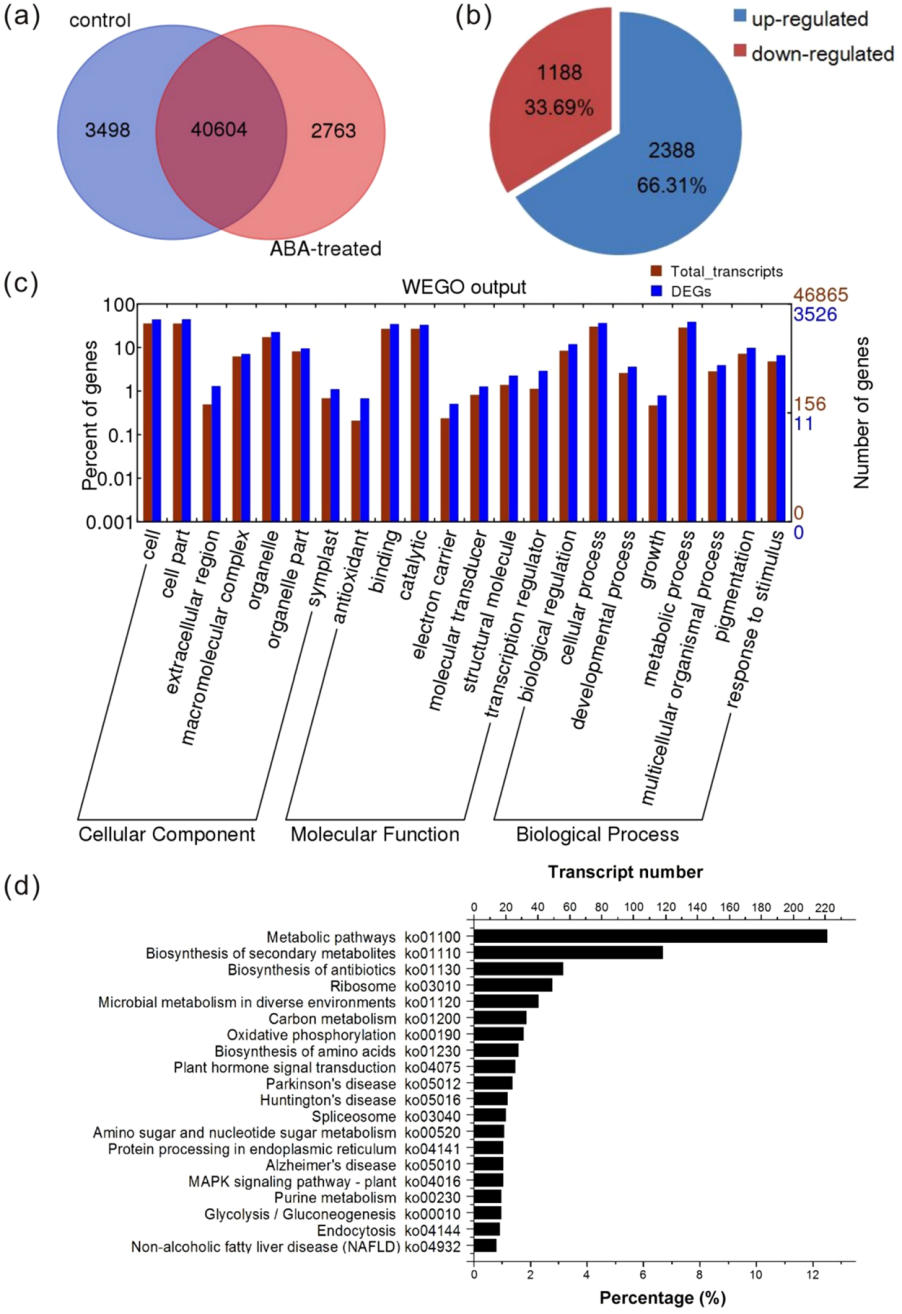
Unigenes significantly differentially transcribed in response to exogenous ABA application. (**a**) Venn diagrams showing the transcript number in the transcriptome of control and ABA-treated group. (**b**) The ABA up-regulated and down-regulated differentially expressed genes. (**c**) Gene Ontology enrichment of the DEGs. (**d**) KEGG analysis of the DEGs (top 20 with significant difference).

A Venn diagram was constructed to present the number of uniquely or commonly expressed genes between control and ABA treatment samples (Fig. 4a). In total, 46,865 transcripts were detected, including 44,102 in the control group and 43,367 in the ABA-treated group. There were 3,498 transcripts uniquely expressed in the control group, and 2,763 transcripts expressed only under the ABA treatment, and 40,604 transcripts commonly expressed in both conditions, which suggested that ABA activated or repressed thousands of particular transcripts during *K*. *obovata* vivipary process.

In order to evaluate the influence of ABA on regulating mangrove vivipary, differential expression analysis was performed by DEseq2^54^ in the comparison between two groups. The transcripts with |log2FC| ≥ 1 and false discovery rate (FDR) <0.05 were identified as differentially expressed genes (DEGs). Our data showed that the expressions of 3,526 transcripts were significantly altered by exogenous ABA, in which 2,338 transcripts were up-regulated, while 1,188 were down-regulated (Fig. 4b and Supplementary Table S3). Among the differentially expressed genes, the number of up-regulated transcripts was larger than down-regulated one, indicating that many genes responded positively to ABA treatment, which was similar to those reported in previous studies in other species^55–57^.

The differentially expressed genes were grouped into GO functional categories to identify the specific biological processes that exogenous ABA altered (Fig. 4c). The DEGs annotated in GO were grouped into terms similar to that of total transcripts on GO.level2. In the comparison between control and ABA-regulated group with p-value < 0.05 defined as significantly enriched GO terms, we found that proteins related to “ developmental process” (GO: 0032502) and “response to stimulus” (GO: 0050896) were significantly enriched terms in the biological process category, and proteins associated with “extracellular region” (GO: 0005576) were enriched in cellular component, while “antioxidant activity” (GO: 0016209) and “transcription regulator activity” (GO: 0030528) were highly represented in the category of molecular function.

Also, the DEGs were subjected to KEGG pathway enrichment analysis, and 1,769 transcripts were assigned to 291 pathways, with 156 pathways significantly different from the background (p-value < 0.05). Our data showed that “metabolic pathways” (ko01100) and “biosynthesis of secondary metabolites” (ko01110) were the most significantly enriched pathways under exogenous ABA treatment. Furthermore, we found that “Plant hormone signal transduction” (ko04075) and “MAPK signaling pathway - plant” (ko04016) pathways were represented significantly (Fig. 4d). In consideration of the facts that ABA signal were usually coupled with MAPK modules^58,59^, this result indicated changes on mangrove ABA signaling regulation. The significantly enriched pathways of DEGs indicated the effects of ABA on mangrove embryo development.

### Differentially expressed genes in plant hormone signal transduction and ABA biosynthesis under exogenous ABA treatment

Besides ABA, other hormones have been proposed to also affect seed dormancy and germination^30,60^. To see more details of the crosstalk of ABA with other hormones during mangrove viviparity, the regulated genes involved in “Plant hormone signal transduction” (ko04075) pathway were highlighted in Supplementary Fig. S1. Our data showed that the transcription levels of many key genes in ABA, auxin, cytokinine, gibberellin and jasmonic acid signal transduction were affected under exogenous ABA treatment.

**Figure 5:**
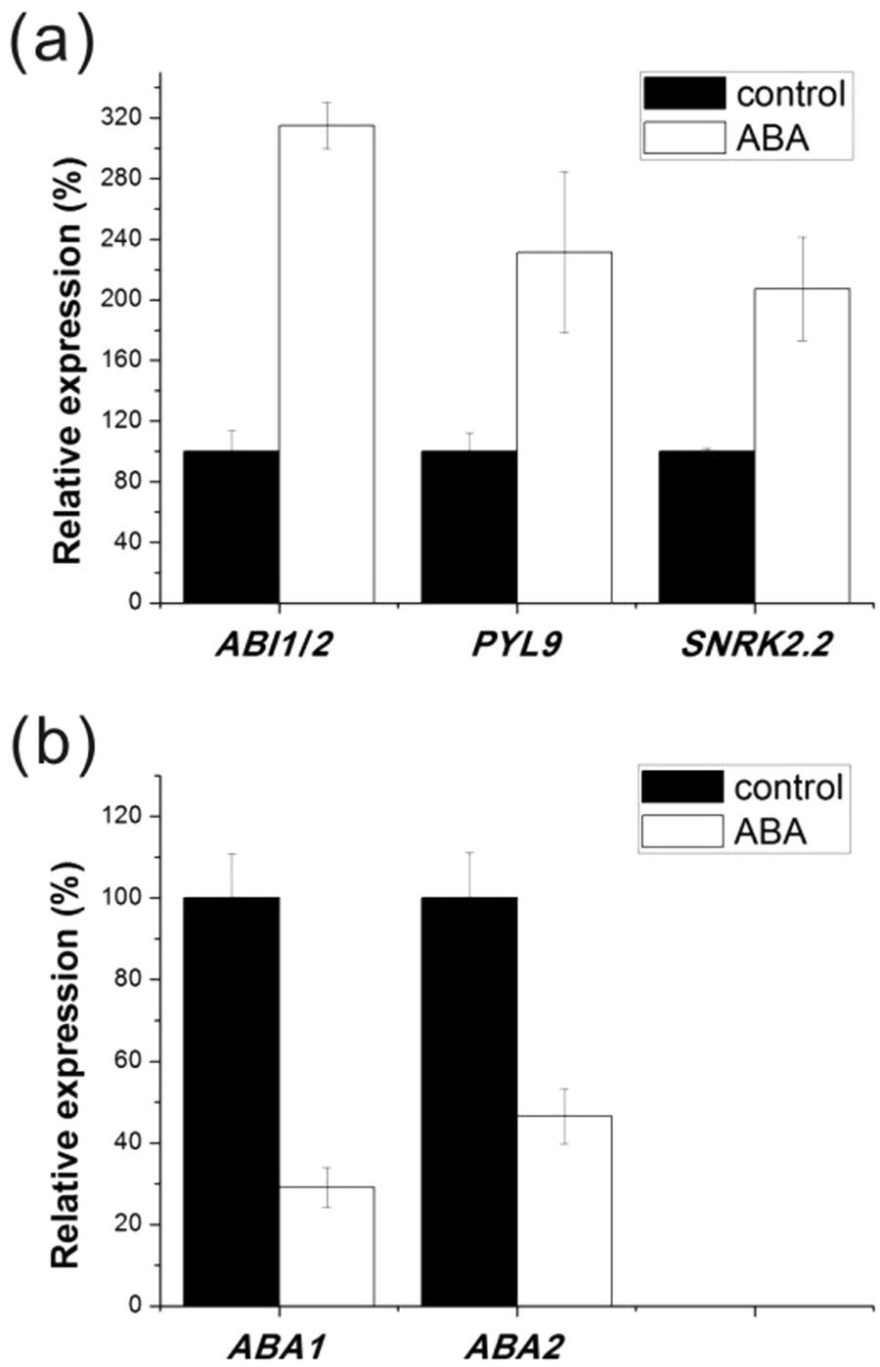
Quantitative RT-PCR analysis of genes involved in ABA signal transduction and biosynthesis pathways. (**a**) Expression of *KoABI1/2*, *KoPYL9* and *KoSNRK2*.*2* response to exogenous ABA. (**b**) Expression of *KoABA1* and *KoABA2* under ABA treatment. The transcript levels of the untreated samples were set to 100%. Data shown are means ± SEM.

To further confirm the RNA-seq results, we analyzed the expression levels of 3 genes participated in ABA pathway by quantitative RT-PCR, including *KoPYL9*, *KoSNRK2*.*2* and *KoABI1/2* (ABI1/2 means that AtABI1 and AtABI2 matched the same protein in this species). We found that the transcript levels of these three genes in ABA signal transduction was dramatically increased by exogenous ABA in *K*. *obovata* (Fig. 5a), consistent with high-throughput sequencing data (Supplementary Table S3).

It has been reported that ABA levels in the embryo of viviparous mangrove was lower than that in non-viviparous^18,35^. Thus, we focus on the transcriptional difference on ABA biosynthesis. Two key enzymes during ABA biosynthesis, *ABA1* (encodes a zeaxanthin epoxidase)^43^ and *ABA2* (encodes a cytosolic short-chain dehydrogenase/reductase)^44^, were significantly induced under ABA treatment in both qRT-PCR and RNA-seq datasets (Fig. 5b and Supplementary Table S3).

### Highly conservative *ABI1* and *ABI2* among Arabidopsis and mangroves

*ABI1* and *ABI2*, which encode group A type 2C protein phosphatases (PP2Cs), act as key factors in a negative feedback regulatory loop of the ABA signal transduction^46,47^. It had been demonstrated that they played a role in seed development and germination^42,48^. In our datasets, it was interesting to find that the transcripts of *ABI1* and *ABI2* were included in DEGs under exogenous ABA treatment as a homologous gene.

**Figure 6:**
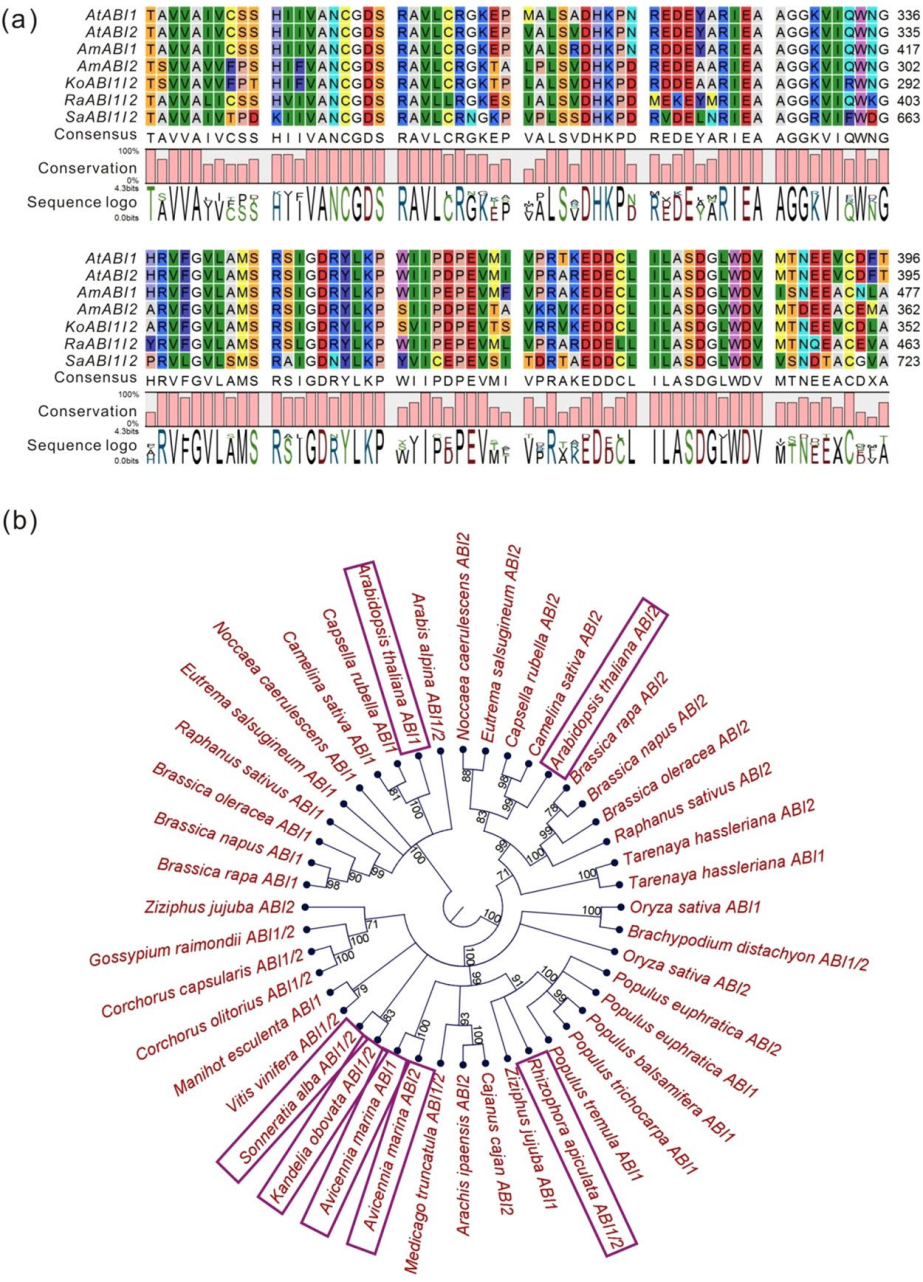
Highly conservative amino acid sequences of *ABI1* and *ABI2* genes among different plants. (**a**) Comparison among the amino acid sequences of AtABI1, AtABI2, SaABI1/2, AmABI1, AmABI2 and RaABI1/2 proteins. (**b**) A phylogenetic tree of the ABI1 and ABI2 proteins. The amino acid sequences of these genes are listed in Supplementary Table S4. Bootstrap threshold (%) >70 was shown.

In order to study their functions on mangrove reproductive process, homologous alignment was performed using amino acid sequences of *ABI1* and *ABI2* in three kinds of mangroves (vivipary, cryptovivipary and non-vivipary) and Arabidopsis. Firstly, we translated the transcriptome of *Sonneratia alba*, *Avicennia marina* and *Rhizophora apiculata* (provided by Prof. Suhua Shi, Sun Yat-sen University, unpublished work). Then, we performed BLAST analysis of amino acid sequences of AtABI1 and AtABI2 in those proteomes, obtaining AmABI1, AmABI2, RaABI1/2 and SaABI1/2 (the only gene that corresponds to both *ABI1* and *ABI2* in other plants). Our results show that *ABI1* and *ABI2* from five species shared high similarity in both amino acid and nucleotide sequences (Fig. 6a and Fig. S2a).

To investigate the evolutionary relationship among *ABI1* and *ABI2* of different plants, a phylogenetic tree was constructed by CLC Genomics Workbench (CLC) software with the full-length amino acid or nucleotide sequences of those genes from NCBI (Fig. 6b and Fig. S2b). It was surprising to find that *SaABI1/2* was separated from *ABI1* and *ABI2* in other species with orthodox seeds, although *S*. *alba* is a non-vivipary mangrove plant. In addition, *RaABI1/2* was clustered with *AmABI1* and *AmABI2* (of a crypto-viviparous plant) rather than with *KoABI1/2*, although *R*. *apiculata* and *K*. *obovata* were viviparous mangroves. Furthermore, SNRK2.2, PYL9, ABA1 and ABA2 were also analyzed with similar results as *ABI1* and *ABI2* (Figs S3–6). These findings strongly indicated that the genes of ABA signaling transduction in mangrove were highly conservative.

### Overexpression of *ABI1* or *ABI2* from Arabidopsis and mangrove show similar ABA Tolerance in transgenic Arabidopsis

To directly investigate their role in ABA signaling, mangrove *ABI1* and *ABI2* genes were introduced into wild-type Arabidopsis plants. Transgenic Arabidopsis lines were generated with the *AmABI1*, *AmABI2*, *RaABI1/2* or *SaABI1/2* gene under the control of CaMV35S promoter. Meanwhile, plants with *AtABI1* or *AtABI2* were generated as positive controls, while seedlings with empty vector as a negative control. Hygromycine resistant plants were confirmed by PCR.

**Figure 7:**
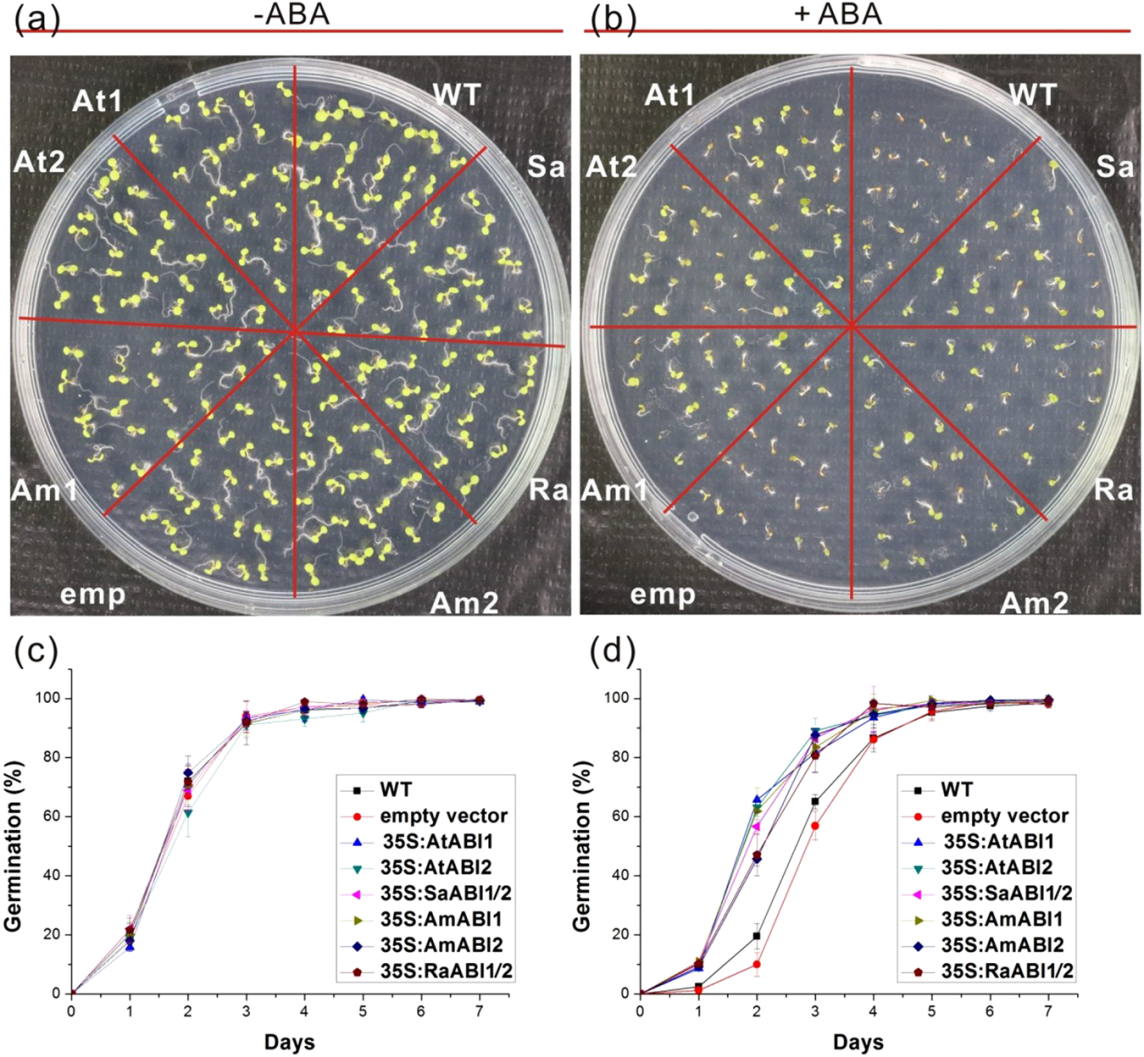
Less sensitivity of the *ABI1* and *ABI2* overexpression lines from mangrove and *Arabidopsis* in response to exogenous ABA. (**a**,**b)** The germination of 7 days wild type (WT), empty vector control (emp) and transgenic *Arabidopsis* seedlings with overexpression of *ABI1* or *ABI2* grown on half MS medium with or without ABA. (**c**,**d**) Germination rates of wild type (WT), empty vector control (emp) and seedlings with overexpression of *ABI1* or *ABI2* grown on medium with or without 0.6 μM ABA. Mean ± SD values were determined from three replicates (n > 200).

In the absence of exogenously supplied ABA, all the transgenic plants germinated as freely as Col-0. However, the germination pattern of the seeds with *ABI1* and *ABI2* gene overexpression similarly resisted to 0.6 µM exogenous ABA in the germination medium, while that with empty vector showed similar sensitivity as wild type as shown in Fig. 7a and b. For examples, as shown in Fig. 7c and d, the germination rate of the six transgenic lines of *AtABI1*, *AtABI2*, *SaABI1/2*, *AmABI1*, *AmABI2* and *RaABI1/2* reached 65.73%, 63.17%, 56.71%, 61.87%, 45.68% and 47.04% respectively, whereas that of Col-0 and empty vector control was 19.49% and 10.01% on the second day after seed were planted. This result indicated that all the genes of *ABI1* and *ABI2* from Arabidopsis and mangroves played similar roles in ABA signaling to promote the growth of the embryo.

## Discussion

The dormancy to germination transition of the seeds is a key physiological process during the life-cycle in almost all seed plants. Unlike orthodox seeds, which experience the desiccation and dormancy^10,11^, mangrove plants disperse propagules with varying degrees of vivipary or embryonic development while the offspring is still attached to the parent tree. Vivipary helps their offspring to survive in the harsh environment. Morphological, ecological and physiological explanations for how vivipary happens are given^61,62^, but little is known about the genetic and molecular mechanism of this process.

ABA was shown to be required to establish seed dormancy in many plants^9,29,30^. Deficiency, insensitivity of ABA and application of ABA inhibitor is associated with reduced dormancy, and sometimes viviparous^25,31–33^. In contrast, exogenous ABA and hypersensitivity of ABA shows increased dormancy and delayed germination^30^. Desiccation is correlated with increased ABA levels^34^. A consistently lower ABA concentration during embryo development of viviparous mangrove has been observed when compared to its non-viviparous relatives^18,35^. However, there is no evidence linking ABA to vivipary directly.

Here, we investigated the influence of ABA on mangrove viviparous process. By directly spraying ABA on fruit of viviparous mangrove, *K*. *obovata*, we found that ABA functions as a negative regulator to repress the fruit growth and development in vivipary process. The inhibitory effect on fruit elongation enhanced with the increase of exogenous ABA concentration. However, ABA application could only delay the growth of *K*. *obovata* fruit, rather than stop mangrove vivipary entirely. This finding suggests that mangrove viviparity is controlled by multiple regulators besides ABA. It would be interesting to reveal the role of other hormones, and their cross-talk with ABA, in terms of viviparous development in mangroves.

Then, we compared the global transcriptome changes between control and ABA-treated group. Our data revealed that thousands of genes expressed differentially under ABA treatment. DEGs involved in the pathways of “Plant hormone signal transduction” were further analyzed, showing that genes participate in many phytohormones were affected (Supplementary Fig. S1), indicating a crosstalk among different phytohormones during mangrove viviparity. Particularly, key genes of ABA biosynthesis, *KoABA1* and *KoABA2*, were repressed in *K*. *obovata* under ABA treatment, although expression levels of genes in ABA signal transduction were induced, including *KoPYL9*, *KoSNRK2*.*2* and ABA negative regulator *KoABI1/2*, demonstrated that there are several feedback regulation pathways of ABA on itself.

Homologous alignment among the amino acid and nucleotide sequences of ABA genes in mangroves with different reproductive modes exhibited that mangrove ABA genes are conservative with the *Arabidopsis*. Evolutionary relationship revealed by a phylogenetic tree among mangrove and other plants further supported that mangroves have an ABA signal transduction pathway highly similar to that in *Arabidopsis*. For example, *SaABI1/2* from non-vivipary mangrove was separated from those in other species with orthodox seeds, and *RaABI1/2* was clustered with crypto-vivipary mangrove *AmABI1* and *AmABI2* rather than with *KoABI1/2*, although *R*. *apiculata* and *K*. *obovata* were of viviparous mangrove group.

Taken together, in normal condition with low ABA concentration in the embryo^18,35^, mangrove offspring directly germinates on the parent tree. After exogenous ABA application, mangroves repressed ABA synthesis by down-regulating key enzymes *ABA1* and *ABA2*, and inhibited ABA signal transduction via an inducement of its negative modulator *ABI1* and *ABI2*. Thus two ABA negative feedback regulatory loops play roles in mangrove viviparous process. The feedback regulation is important for mangrove to survive, because ABA accumulation would be induced by environmental stimuli, such as salt, oxidative stress and high temperature^36–39^.

## Materials and Methods

### ABA field application experiment

To assess the effect of exogenous ABA on fruit development of viviparous mangrove, the ABA application experiment was conducted using plants of *K*. *obovata* at Yundang Lake, Xiamen, Fujian province, China (118°10′N, 24°48′E). For this, branches from three *K*. *obovata* trees were randomly chosen for the ABA spray experiment of an individual repeat. Four different treatments were performed to branches of each tree (one branch for each treatment): (I) a control treatment of 0.5% (v/v) Tween-20; (II) 40 μM ABA in 0.5% (v/v) Tween-20 (the same on other treatments); (III) 200 μM ABA and (IV) 500 μM ABA. The treatments were replicated on separate plants at least three times (totally nine trees). The branches were sprayed until runoff with a hand-held sprayer in the late afternoon once per week continuously for 6 weeks (replenish if rain). Experiments were performed mainly July - September in 2015, and repeated in 2016.

### RNA extraction

For transcriptome sequencing, twelve fruits were taken randomly from the sprayed branches. Sampled fruits were washed in 70% ethanol and rinsed twice in sterile water. After peeling off the fruit coats, samples were kept in the RNA storage reagent RNAlater (Ambion) at 4 °C for 24 hours for permeation, and finally stored at −80 °C until RNA isolation. RNA extraction was performed using MiniBEST Plant RNA Extraction Kit (Takara) according to the instruction manual. RNA concentration and purity analysis was checked by Nanodrop 2000 and Agilent Bioanalyzer 2100. Each treatment had 3 replicates.

### RNA-seq library construction and sequencing

Global transcriptome analysis was profiled using RNA isolated from embryos with or without ABA treatment, using a method similar to previously described^63^. For each sample, equal quantities of high-quality RNA from 12 fruits were pooled for cDNA library construction. Briefly, 2 µg DNA-free total RNA from each sample were purified via oligo(dT)25 magnetic beads (Invitrogen), and then fragmented in 5 × first strand buffer (Invitrogen) at 94 °C for 2 min. The cleaved RNA fragments were reverse-transcribed into the first-strand cDNAs by 3′ and 5′ adaptors, followed by 18 cycles of PCR amplification with Illumina adapters with Phire II (Thermo Fisher Scientific). The library was run on an agarose gel, and a 300 to 500 nt band was excised and purified by using QIAquick gel extraction kit (Qiagen). The final PAT-seq libraries were quality checked by Qubit and Agilent Bioanalyzer 2100 before Illumina HiSeq 2500 sequencing was performed at an in-house facility at the College of the Environment and Ecology, Xiamen University. All RNA-seq read files are available from the NCBI Sequence Read Archive (SRA) (http://www.ncbi.nlm.nih.gov) (accession number SRP107989).

### De novo assembly of RNA-seq data

For quality control, raw data of Fastq format were processed through the Trimmomatic_0.36^64^ and Fastx-toolkit^65^ to eliminate the low quality reads (reads containing sequencing adapters; nucleotide with q quality score lower than 20 or reads containing poly(N) (unknown sequence) >5), resulting in clean reads. Then the high quality clean data was used for further analyses.

The pipeline of *de novo* transcriptome assembly and analysis used in this study was described previously^66^. Briefly, read1 and read2 from all libraries were put together to form one left.fq and right.fq file, respectively. Then, these two files were subject to analysis by Trinity with min_kmer_cov 3 and other default parameters.

### Differential expression analyses

The DESeq2 package^67^ was used to search for differentially expressed poly(A) clusters. DESeq2 estimates the variance of expression levels based on read number. The p-values were adjusted using Benjamini-Hochberg multiple testing corrections^68^.

### Gene Ontology (GO) and Kyoto encyclopedia of genes and genomes (KEGG) enrichment analyses

All the genes of *K*. *obovata* with GO terms for biological processes, molecular functions, and cellular components classification were annotated by Blast2GO vision 3.3 with default parameters^53^, and analyzed by WEGO^69^. We analyzed the functional pathway for each gene of *K*. *obovata* by KEGG database from the online server (http://www.genome.jp/tools/kaas/)^70–72^. Differentially expressed genes were also subjected to GO and KEGG pathway enrichment analyses, and those groups with p-value < 0.05 were defined as significantly enriched GO terms or KEGG pathways.

### Quantitative real-time PCR

Reverse transcription was carried out with 2 µg DNA-free total RNA using first-strand cDNA synthesis kit (Invitrogen Inc.). Quantitative RT-PCR assay was performed using the CFX96™ Real-Time PCR Detection System (Bio-Rad, Inc.) with SYBR green PCR master mix. *KoUBQ1* and *KoACT2* were used as reference genes. PCR was performed as follows: 3 min at 95 °C, followed by 40 cycles of denaturation for 10 s at 95 °C, annealing for 10 s at 55 °C, and extension for 20 s at 72 °C. All experiments were performed with three independent biological replicates and three technical repetitions. The specific primers of those analyzed genes are listed in Supplementary Table S1.

### Phylogenetic analysis

The multiple comparisons were performed to evaluate the levels of similarity and difference among nucleotide and amino acid sequences of target genes using the CLC Genome Workbench. Phylogenetic evaluation used the maximum likelihood method with 1,000 bootstrap simulations, conducted with the CLC Genome Workbench and MEGA5 software^73^. Bootstrap values greater than 70% were regarded significant.

### Plant material, growth conditions and chemical treatments

*Arabidopsis thaliana* ecotypes Columbia (Col-0) was used as wild-type (WT). Seeds were surface-sterilized for 5 min in 5% commercial kitchen bleach, washed three times with sterile water, and plated on half-strength Murashige and Skoog medium (pH 5.8, 1% sucrose and 1% agar) with or without ABA. Plants were stratified at 4 °C for 3 days in the darkness and then transferred to a phytotron set at 23 °C under the light intensity of 80 mM photons m-2s-1 in horizontally oriented Petri dishes. Photoperiod was 16 hours light/8 hrs dark cycles (16 L/8D). Seedlings were analyzed at 7 days after germination.

### Vector constructs and transgenic lines

Coding regions of Six *ABI1*/2 homologous genes from all four species (*Arabidopsis thaliana*, *Sonneratia alba*, *Avicennia marina* and *Rhizophora apiculata*) was PCR amplified and inserted into the *Sma*I sites of the binary vector pCAMBIA1300 under the control of a 35 S promoter (http://www.cambia.org), resulting in transcriptional fusion of the 35 S promoter with the *ABI1*/2 coding region. This construct was sequenced and transformed into *Arabidopsis* Col-0 by *Agrobacterium tumefaciens* strain pGV3101 using the floral-dip method^74^. Primer sequences for *ABI1/2* homologous genes are listed in Supplementary Table S1.

For the screening of transgenic lines carrying the empty vector (control) and 35S:*ABI1*/2, T1 seedlings were grown in half-strength Murashige and Skoog medium containing 50 µg/mL hygromycin B (Invitrogen) for 10 days, and the positive plants were transferred to soil for further growth. More than 16 independent T2 lines were obtained for each transgene.

## Electronic supplementary material

Supplementary Information

Supplemental Table S3 List of differential expressed genes

Supplemental Table S4 The amino acid sequences used for the phylogenetic tree
