# Supplementary material for: Transcriptome profiling during mangrove viviparity in response to abscisic acid
Source: Sci Rep. 2018 Jan 15;8:770. doi: 10.1038/s41598-018-19236-x (PMC5768736; doi:10.1038/s41598-018-19236-x)
Supplement: Supplementary file 1 — Supplementary Information [file 41598_2018_19236_MOESM1_ESM.pdf]

Transcriptome profiling during mangrove viviparity in response to abscisic acid

Liwei Hong[1], Wenyue Su[1], Yuanye Zhang[1], Congting Ye[1], Yingjia Shen[1], Qingshun Q. Li[1,2*]

Supplementary Materials

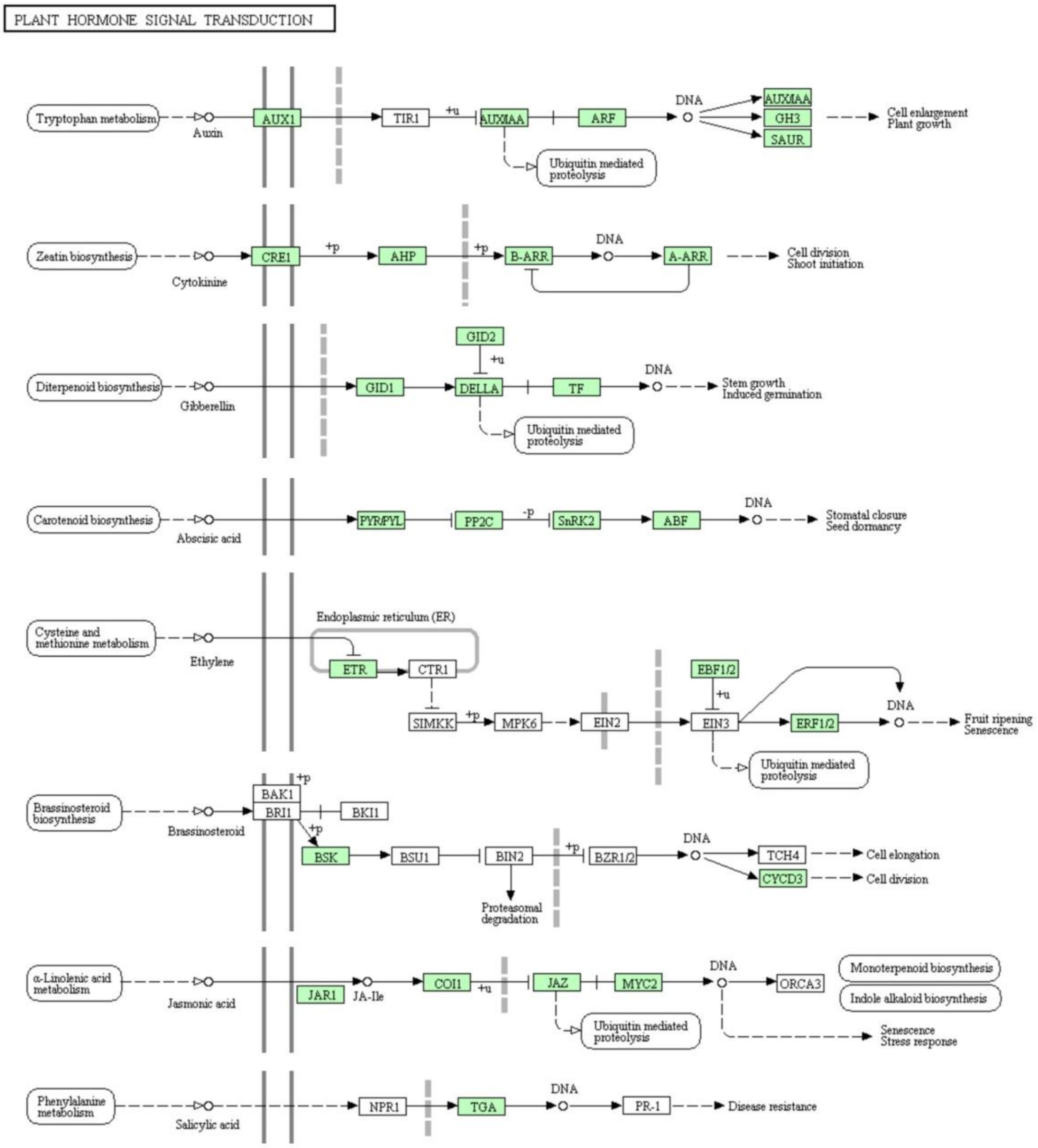

Supplementary Fig. S1. Differential expression genes involved in “Plant hormone signal transduction” response to exogenous ABA application (including auxin, cytokinine, gibberellin, abscisic acid, ethylene, brassinosteroid, jasmonic acid and salicylic acid pathway) using an automatic genome annotation and pathway reconstruction server KAAS[1].

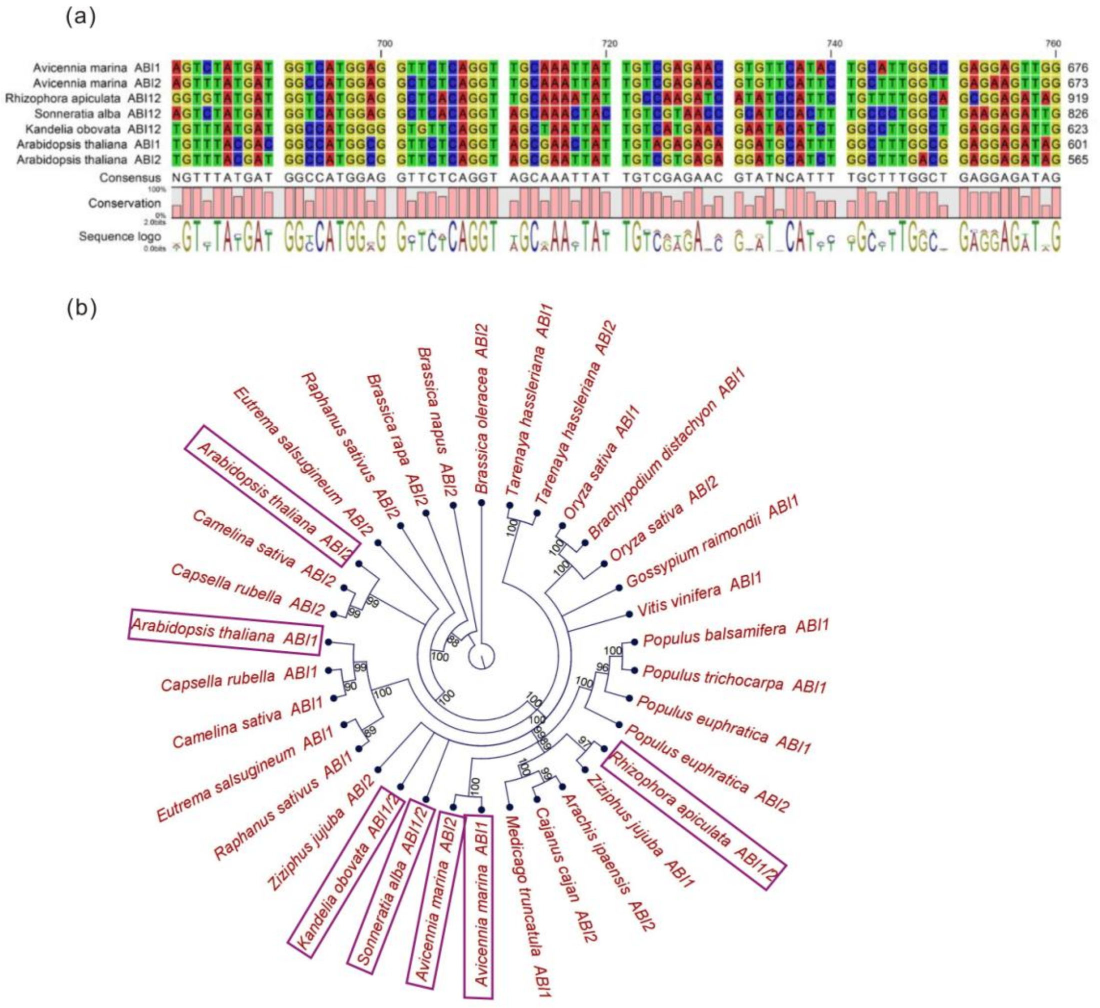

Supplementary Fig. S2. Highly conservative nucleotide sequences of ABI1 and ABI2 genes among different plants. (a) Comparison among the nucleotide sequences of *AtABI1*, *AtABI2*, *SaABI1/2*, *AmABI1*, *AmABI2* and *RaABI1/2*. (b) A phylogenetic tree of the *ABI1* and *ABI2* DNAs. Bootstrap threshold (%) > 70 was shown.

(a)

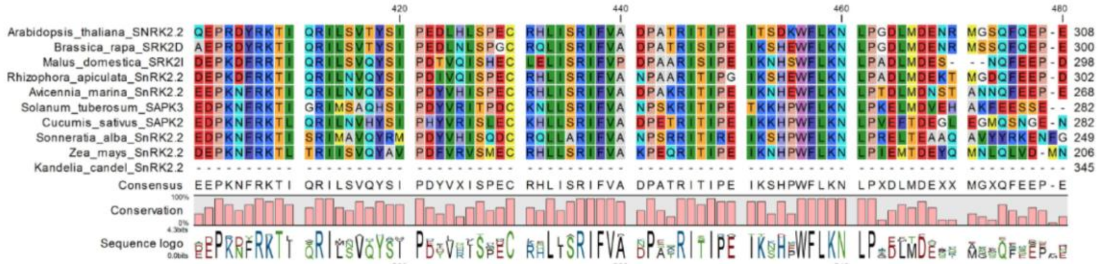

(b)

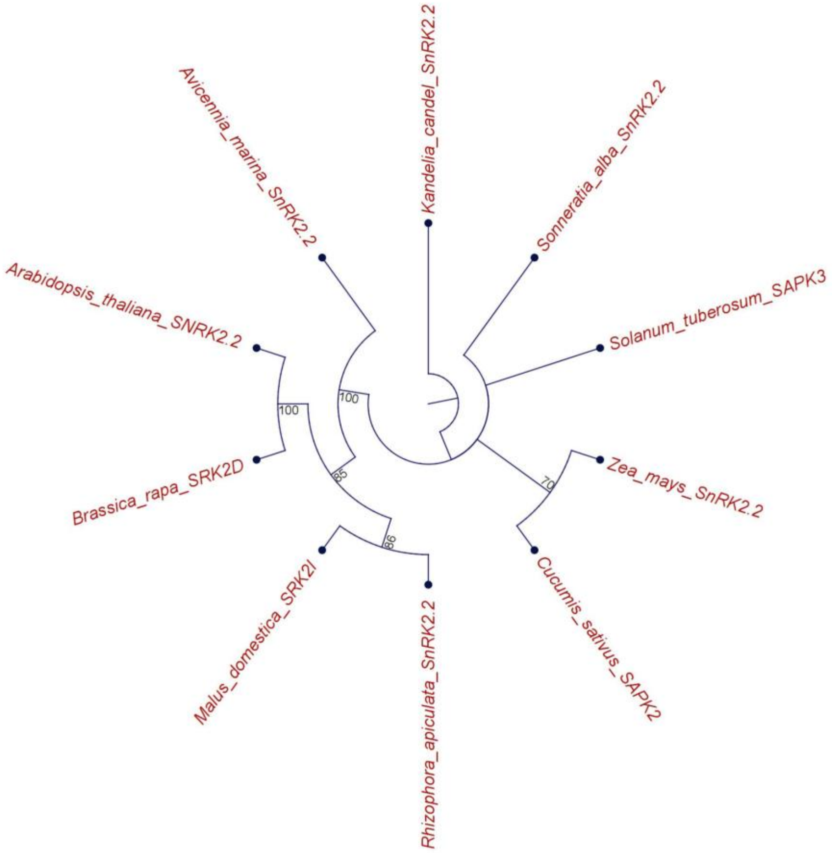

Supplementary Fig. S3. Highly conservative amino acid sequences of SNRK2.2 genes among different plants. (a) Comparison among the amino acid sequences of SNRK2.2 proteins. (b) A phylogenetic tree of the SNRK2.2 proteins. Bootstrap threshold (%) > 70 was shown.

(a)

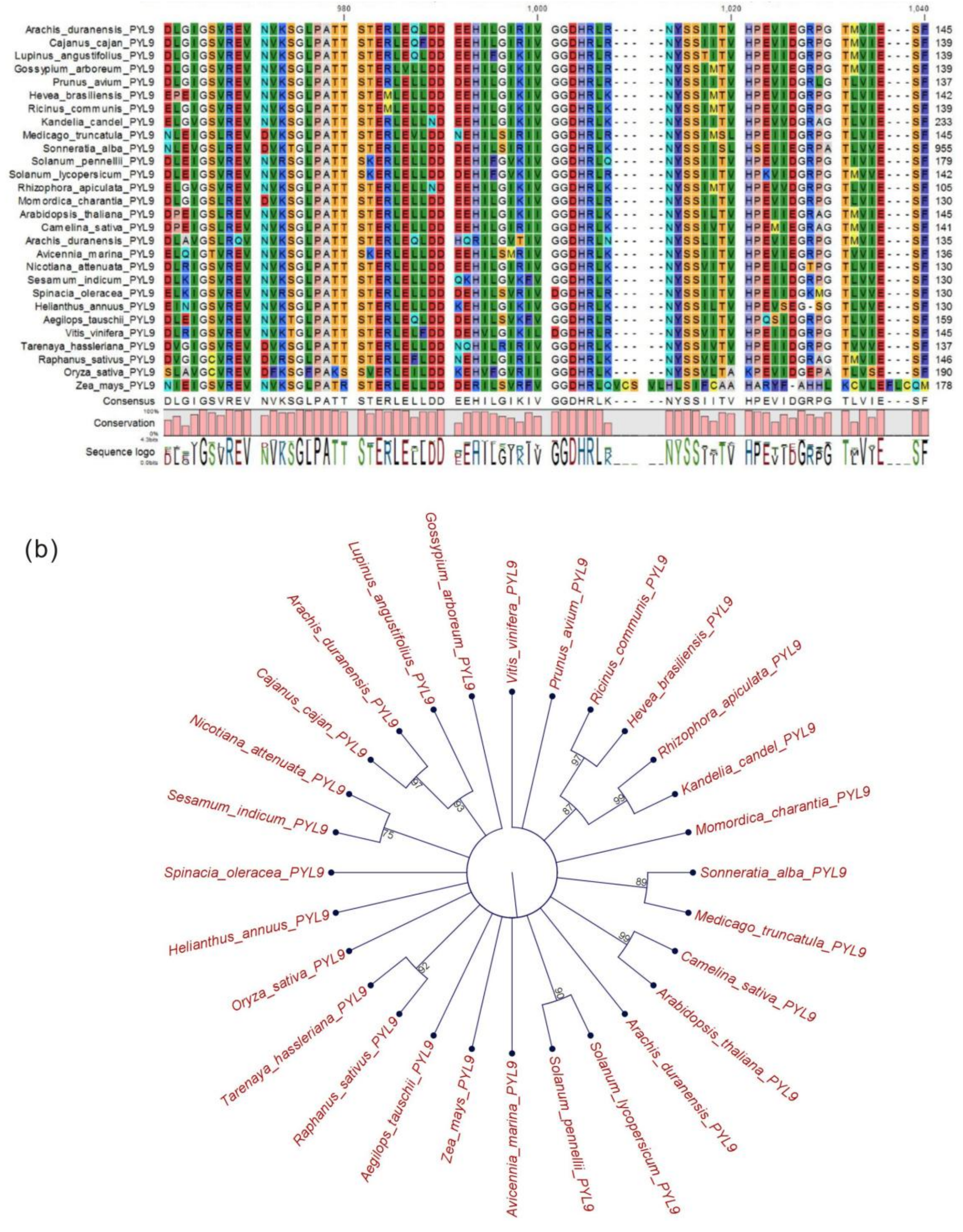

Supplementary Fig. S4. Highly conservative amino acid sequences of PYL9 genes among different plants. (a) Comparison among the amino acid sequences of PYL9 proteins. (b) A phylogenetic tree of the PYL9 proteins. Bootstrap threshold (%) > 70 was shown.

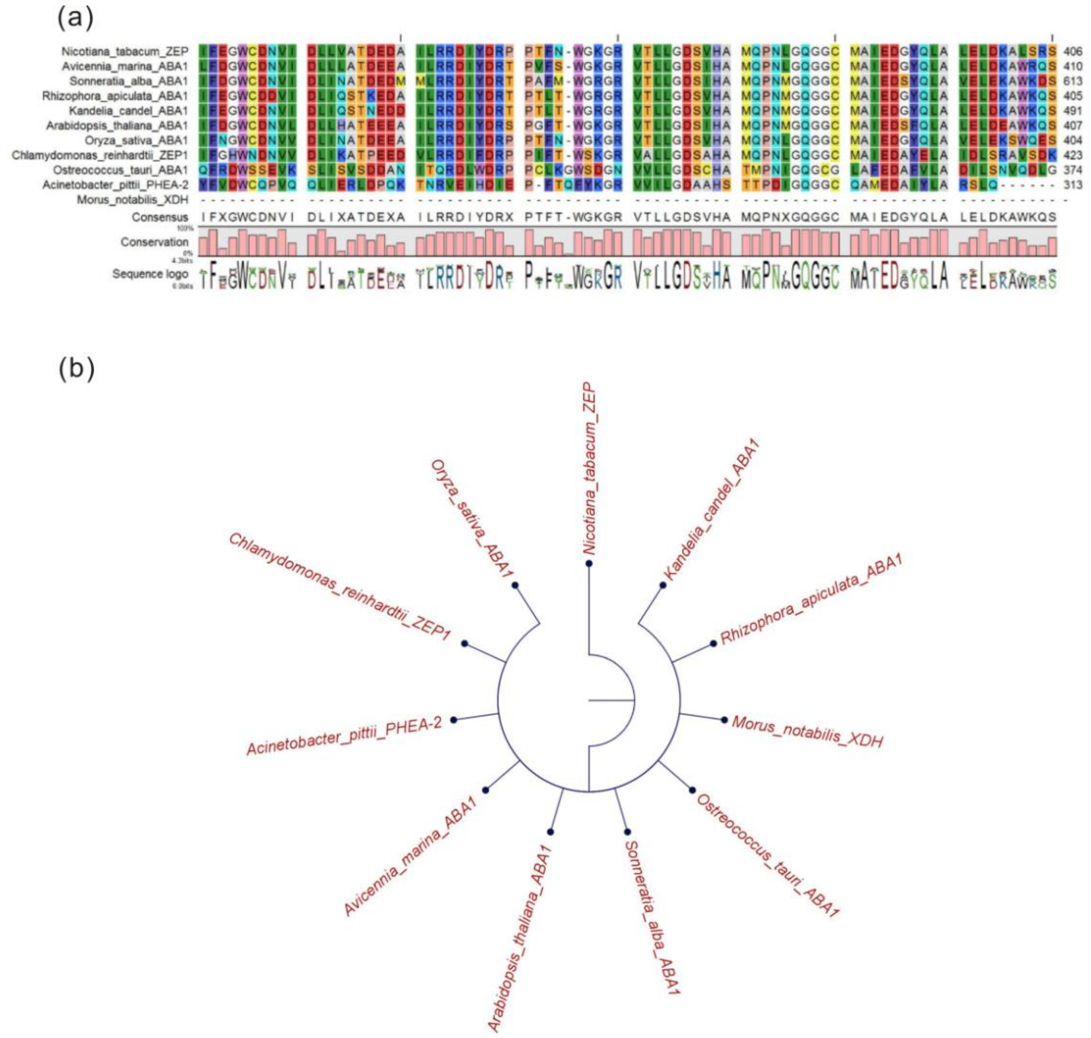

Supplementary Fig. S5. Highly conservative amino acid sequences of ABA1 genes among different plants. (a) Comparison among the amino acid sequences of ABA1 proteins. (b) A phylogenetic tree of the ABA1 proteins. Bootstrap threshold (%) > 70 was shown.

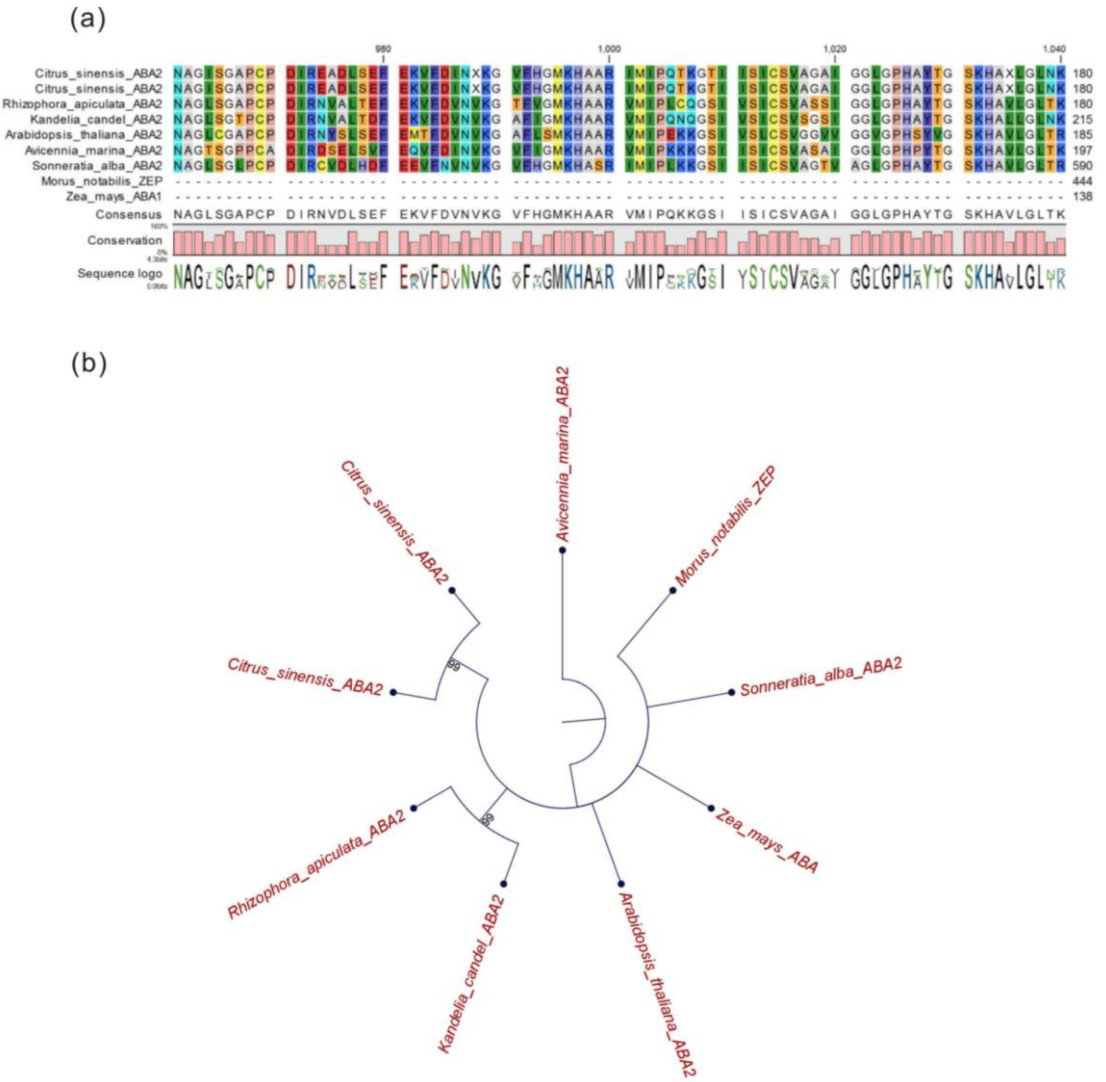

Supplementary Fig. S6. Highly conservative amino acid sequences of ABA2 genes among different plants. (a) Comparison among the amino acid sequences of ABA2 proteins. (b) A phylogenetic tree of the ABA2 proteins. Bootstrap threshold (%) > 70 was shown.

Supplementary Table S1. List of primers used in this study.

ATCTTTACCGTTAATGGAGGAAG	AtABI1-F
CCTCTCTGCCTCAGTTCAAG	AtABI1-R
TAATGGACGAAGTTTCTCCTGC	AtABI2-F
TTTCAATTCAAGGATTTGCTCTTG	AtABI2-R
ATGCTGGCTATGACTTCCGAG	AmABI1-F
CTACGTTCTGCTCTTGATCTTTC	AmABI1-R
ATGGGTGATCCAGCAAGCTC	AmABI2-F
TTAAGGCTGAGTTCTGCTCCTG	AmABI2-R
ATGTACCGGGCAGTTGCAG	raABI12-F
TCAAGTTTTGCTCTTGAACTTCC	raABI12-R
ATGGAAGATATGTCCCCATCTG	saABI12-F
TCAGGACTTGGTCTTGAACTTTC	saABI12-R
ACCGAGGCTCCTCTTAATCC	koACT2-qrtF
AGCTGGCACATTGAAGGTCT	koACT2-qrtR
AAGGCCAAGATCCAAGACAA	koUBQ1-qrtF
CTGCTTGCCAGCGAAGAT	koUBQ1-qrtR
TTGGTGCTGATGGAATATGG	koABA1-qrtF
AGCAAGTGTAGCCCGAGTAAA	koABA1-qrtR
ACTTGTCTTTGGCTCACTTGC	koABA2-qrtF
GAAAACCTGCCATTGCATC	koABA2-qrtR
GTCGCCATCGTCACACCT	koABI12-qrtF
GACGGCTCTGGAGTCACC	koABI12-qrtR
CACCTGTTCACCTTGTCTGG	koPYL9-qrtF
ACCTGTTCACAAATGGCTTGT	koPYL9-qrtR
GACTTGGCAATTGTCATGGAA	koSNRK2.2-qrtF
GCCAGCACTGCATATCCTTC	koSNRK2.2-qrtR

Supplementary Table S2. Data from raw to clean sequences.

sample	rawdata		cleandata	
	number	amount(Gb)	number	amount(Gb)
control-1read1	19625832	5.54	13913106	3.56
control-1read2	19625832	5.54	13913106	3.41
control-2read1	14334561	4.05	10321789	2.66
control-2read2	14334561	4.05	10321789	2.55
control-3read1	12707930	3.59	9459647	2.44
control-3read2	12707930	3.59	9459647	2.34
ABA-1read1	25932244	6.55	23900201	5.64
ABA-1read2	25932244	6.55	23900201	5.64
ABA-2read1	23333589	5.89	22279160	5.25
ABA-2read2	23333589	5.89	22279160	5.25
ABA-3read1	17639369	4.45	16370368	3.86
ABA-3read2	17639369	4.45	16370368	3.86
total	227147050	60.14	192488542	46.46

Reference

1. Moriya, Y., Itoh, M., Okuda, S., Yoshizawa, A. & Kanehisa, M. KAAS: an automatic genome annotation and pathway reconstruction server. Nucleic Acids Res. 35, W182-W185 (2007).
